# An IROA Workflow for correction and normalization of ion suppression in mass spectrometry-based metabolomic profiling data

**DOI:** 10.21203/rs.3.rs-3914827/v1

**Published:** 2024-02-01

**Authors:** Iqbal Mahmud, Bo Wei, Lucas Veillon, Lin Tan, Sara Martinez, Bao Tran, Alexander Raskind, Felice de Jong, Rehan Akbani, John N. Weinstein, Chris Beecher, Philip L. Lorenzi

## Abstract

Ion suppression is a major problem in mass spectrometry (MS)-based metabolomics; it can dramatically decrease measurement accuracy, precision, and signal-to-noise sensitivity. Here we report a new method, the IROA TruQuant Workflow, that uses a stable isotope-labeled internal standard (IROA-IS) plus novel companion algorithms to 1) measure and correct for ion suppression, and 2) perform Dual MSTUS normalization of MS metabolomic data. We have evaluated the method across ion chromatography (IC), hydrophilic interaction liquid chromatography (HILIC), and reverse phase liquid chromatography (RPLC)-MS systems in both positive and negative ionization modes, with clean and unclean ion sources, and across different biological matrices. Across the broad range of conditions tested, all detected metabolites exhibited ion suppression ranging from 1% to 90+% and coefficient of variations ranging from 1% to 20%, but the Workflow and companion algorithms were highly effective at nulling out that suppression and error. Overall, the Workflow corrects ion suppression across diverse analytical conditions and produces robust normalization of non-targeted metabolomic data.

Metabolite levels reflect metabolic function and the integrated output of genomics, epigenomics, transcriptomics, and proteomics, including inputs from lifestyle and environment[1–3]. Hence, metabolomics is an effective approach for elucidating candidate drug targets[4], candidate biomarkers of disease progression[5], candidate biomarkers of therapeutic response[6,7], mechanism(s) of drug sensitivity[8], mechanism(s) of drug resistance[9], and mechanisms of drug toxicity[10]. Unfortunately, rigorous, reproducible quantitation of metabolites is difficult. Standardization across laboratories, biological matrices, and analytical conditions is a major challenge for both research and clinical implementation of metabolomics.

Ion suppression is a type of matrix effect in mass spectrometry (MS) and a major contributor to those challenges (**Fig. 1**). The authors of a recent perspective article on best practices in metabolomics noted: "While there is no universal solution to the ion suppression problem, assessing the effects of ion suppression affords greater confidence in the accuracy of the results."[11] Indeed, until now, no universal solution has existed to counteract the negative effects of ion suppression across all analytes in a non-targeted metabolite profiling study. We present such a solution.

The mechanisms of ion suppression (e.g., in plasma, urine, cell culture, or tumor) are reviewed in detail elsewhere[11–13]; the type of ionization source[5,14–17], mobile phase composition[18], gas temperature, and physicochemical properties (e.g., pKa, polarity/aromaticity, hydrophobicity/ lipophilicity) of analytes and matrix components are examples of factors that can contribute to ion suppression[12,19,20].

Ion suppression for small numbers of analytes (e.g., in targeted MS) can be addressed to some degree by diluting samples, modifying chromatographic or MS conditions to eliminate interferences, conducting a sample cleanup procedure such as solid phase extraction, and/or adding a chemically matched stable isotope-labeled internal standard[11,21]. However, because the source and magnitude of ion suppression can vary extensively across metabolites and samples[11,22], counteracting ion suppression across all analytes and all samples in a non-targeted profiling study remains an unsolved challenge[11,23].

Stable isotope-labeled internal standards can correct for variability in ionization efficiency and ion suppression. However, isobaric isotopologs (e.g., the M+0 isotopolog of lactate and the M+1 isotopolog of alanine) are difficult to distinguish. That has been a barrier to the effective use of stable isotope mixtures. Isotopic Ratio Outlier Analysis (IROA) protocols[24–27] solve that problem by generating clearly identifiable isotopolog patterns. IROA also facilitates removal of non-biological signals, which are common artifacts in MS data.

We introduce here a novel Workflow that effectively corrects ion suppression and uses a Dual-MSTUS (MS Total Useful Signal) normalization algorithm to improve the quantitative accuracy, precision, and signal-to-noise sensitivity of metabolomic data across diverse origins and analytical conditions.

## Results

**Conceptual Overview.** The Workflow presented here is based on the IROA Internal Standard (IROA-IS) and a chemically identical but isotopically different Long-Term Reference Standard (IROA-LTRS) (**Fig. 2**). The Workflow identifies each molecule in any type of sample based on a unique, formula-specific isotopolog ladder (**Fig. 2C′, D′**) created by (i) a low $^{13}C$ (natural abundance or 5%) signal from the isotopologs of the molecule in question at the low mass end of the ladder and (ii) a 95% $^{13}C$ signal for the isotopologs at the high mass end of the ladder. It is useful to call the collection of lower mass peaks in the isotopolog ladder the 12C channel and the collection of higher mass peaks in the ladder the 13C channel. IROA-LTRS (**Fig. 2D**, red/yellow samples) is a 1:1 mixture of chemically equivalent IROA-IS standards at 95% $^{13}C$ and 5% $^{13}C$. The combination produces the IROA-LTRS isotopic pattern illustrated in **Fig. 2D′**. Metabolite $^{12}C$ and $^{13}C$ isotopologs co-elute, and the signature IROA peak pattern distinguishes real metabolites from artifacts, which lack the IROA pattern.

Since metabolites in the Internal Standard are spiked into samples at constant concentrations, the loss of $^{13}C$ signals due to ion suppression in each sample can be determined and used to correct for the loss of corresponding $^{12}C$ signals. To model ion suppression, we created a single methanol extract of plasma, divided it into aliquots ranging from 50 to 1,500 µL, dried the aliquots, and reconstituted them with a fixed volume and concentration of IROA-IS. We developed **Equation 1** to calculate and correct % ion suppression as described in Methods.

Biologically relevant signals are considered to be those observed in both the IROA-LTRS and analytical samples as an IROA signature isotopolog ladder of signals with regular M+1 spacing, decreasing amplitude signal in the $^{12}C$ channel, and increasing amplitude signal in the $^{13}C$ channel (from low to high mass) (**Fig. 3A**). An initial goal of the Workflow for quantitative metabolomic profiling is to calculate an AUC-$12C_{suppression\text{-}corrected}$ value for each metabolite (**Equation 1**). Without ion suppression, endogenous ($^{12}C$) metabolite AUCs should increase linearly with aliquot volume, and internal standard ($^{13}C$) levels should remain constant (**Fig. 3B**). However, the MSTUS values[28] calculated for both [MSTUS-12C] (blue lines) and [MSTUS-13C] (green lines) deviate from proportionality because of increasing ion suppression with increasing sample concentration. Nevertheless, because ions in the $^{12}C$ and $^{13}C$ isotopolog channels experience equal degrees of suppression with increasing sample volume, IROA-IS enables accurate quantitation and correction of ion suppression for each metabolite (**Equation 1**).

**Ion suppression correction in non-targeted metabolomics.** As expected, IROA-IS-based suppression-corrected values for MSTUS-13C were constant across sample input volumes, and $^{12}C$ values increased proportionally to sample input (**Fig. 3B**). To explore the generalizability of the IROA Workflow, we performed a detailed characterization of ion suppression in both positive and negative ion modes for three highly used chromatographic systems: IC-MS, RPLC-MS (C18-MS), and HILIC-MS with unclean and clean electrospray ionization (ESI) source conditions (**Fig. 4A**). Negative ionization mode detected fewer ions than did positive ionization mode (**Fig. 4B**), but extensive ion suppression was evident in both polarities and all chromatographic systems. Ion suppression calculation and corrections were performed automatically by ClusterFinder software (version 4.2.21, 64-bit, IROA Technologies) using **Equation 1**. The list of metabolites present in all samples in each chromatographic system (the Dual MSTUS Metabolite list) is provided in **Supplementary Table 1-5**. As expected, uncleaned ionization sources demonstrated significantly greater levels of ion suppression than cleaned ionization sources (**Fig. 4C-K**).

Strikingly, all tested chromatographic systems and conditions exhibited up to nearly 100% ion suppression, which was effectively corrected by the IROA Workflow, which yielded a linear increase in signal with increasing sample input (**Fig. 4L, Supplementary Table 6, and Supplementary Fig. 1, 2**). Of course, if a metabolite peak were 100% suppressed in the $^{12}C$ or $^{13}C$ channel, it would not yield the IROA ladder pattern and, therefore, would appear fully suppressed and absent from the output. But even if we attempted to include such a metabolite, the additional unknown in **Equation 1** would prevent its correction. Hence, the greatest weakness of the Workflow is that the output includes only metabolites that are detected in both the $^{12}C$ and $^{13}C$ channels (i.e., at most 99% suppressed). Absence from the $^{12}C$ or $^{13}C$ channel could be caused by 100% suppression, true absence in the IROA-IS, or true absence from the sample. Future versions of the ClusterFinder software will facilitate identification of fully suppressed analytes.

Historically there has been a tradeoff between injecting a large sample volume to increase assay sensitivity and injecting a smaller volume to decrease matrix effects. Using this Workflow, analysts can err on the side of injecting larger sample volumes to ensure robust measurement of low-abundance analytes while simultaneously performing ion suppression correction to achieve more accurate results. The Workflow produces accurate concentration values for most analytes, even in highly concentrated samples. For example, tryptophan (M+H -NH3) exhibited 8% ion suppression in RPLC positive mode with a cleaned ionization source, and suppression correction restored the expected linear increase in signal with increasing sample input (**Fig. 4M**). In a more extreme example, pyroglutamic acid exhibited up to 92% suppression in IC-MS negative mode, and the IROA workflow corrected for that suppression as well (**Fig. 4N**).

The Workflow facilitated identification and measurement of 539 different metabolites across the entire sample set (**Fig. 4O**). An average of 422 metabolites were observed in each sample, and 216 were common to all samples. Unexpectedly, the total number of peaks observed decreased as a function of sample input, likely due to the suppression driving some compounds' peaks to extinction. Kohonen Self Organizing Maps (SOMs) revealed patterns of increasing concentration with sample input, decreasing concentration with sample input, and various intermediate patterns (**Figs. 4P, Q and Supplementary Fig. 3**). For instance, **Figs. 4P-15, 4P-13,** and **4P-09** indicate metabolite peaks that first increase and then decrease in amplitude as the input volume is increased. Analysis of the full dataset shows: i) for suppression levels up to 60%, the observed [AUC-$12C_{suppressed}$] value was weakly correlated with the expected [AUC-$12C_{suppression\text{-}corrected}$] value; from 60% to 80% suppression, the correlation was lost; and above 80% suppression, [AUC-$12C_{suppressed}$] values were negatively correlated with the expected [AUC-$12C_{suppression\text{-}corrected}$] value due to decreasing peak size with increasing sample input.

We next evaluated the performance of IROA-based ion suppression correction with urine samples, which notoriously exhibit high ion suppression[5,16]. We were pleased to find that the IROA Workflow produced comparable suppression-corrected data in both positive and negative ionization modes (**Figs. 4R, S**). The correction was robust even at high urine sample inputs for which the ion suppression effects were most severe (**Fig. 4T** and **Supplementary Fig. 4**). Common metabolites identified in both plasma and urine revealed that ion suppression correction yielded comparable results in the two matrices (**Fig. 4U**). Overall, the ability of the IROA Workflow to produce a linear increase in suppression-corrected signals consistently with increasing sample input demonstrates the broad applicability and versatility of the Workflow for ion suppression correction across diverse biofluids. Moreover, our results demonstrate that the Workflow successfully corrects ubiquitous ion suppression across diverse metabolic pathways, analytical techniques, and experimental conditions (**Fig. 5A, B, & C and Supplementary Fig. 5**).

**Development of Dual MSTUS for robust normalization of metabolomic data.** The Workflow presented in the previous section promises to improve quantitative accuracy and correct for a subset of factors that introduce variation in metabolomic assay sensitivity, but ion suppression correction alone cannot enable accurate comparison of metabolite levels across variable sample input, analytical batches, biological matrices, instruments, and many other factors. Inconveniently, accurate cross-matrix comparison requires absolute quantitation using matrix-matched external calibration curves. Dual MSTUS normalization provides a solution to those challenges.

MSTUS normalization determines a total useful signal value for each sample by excluding all clearly non-relevant signals and then sums the remaining data[22]. That relatively simple strategy has been successful in metabolomics. Here we extend the MSTUS strategy into a Dual MSTUS algorithm. Since the IROA-IS $^{13}C$ values for each metabolite should be equal across all samples, adjusting each MSTUS-12C value to the corresponding MSTUS-13C value normalizes all $^{12}C$ signals to produce accurate comparisons across variable conditions (**Fig. 6**). An important distinction here is that, unlike suppression correction, wherein each compound is corrected individually, MSTUS normalization calculates a Normalization Factor, NF, that is applied to all peaks in each sample (see Methods for detail).

When applied to the data presented in the previous section, Dual MSTUS normalization produced significantly improved precision across different chromatographic methods and conditions (**Fig. 6A-K**). Notably, metabolites lacking a matched IROA-IS could not be suppression-corrected but still benefited from Dual MSTUS normalization because NF is a sample-based value and not a metabolite-specific value (**Supplementary Fig. 6 and Supplementary Table S7**). Since those measurements were not corrected for ion suppression, the normalized data still reflected suppressed signal, but Dual MSTUS normalization improved the biological and statistical significance of comparisons across samples, analytical conditions, and matrices with dramatic decrease in error from 16% to below 1% in plasma and from 50+% to below 1% in urine (**Fig. 6L and Supplementary Table S8**). Note that we designed the experiment to include extreme variance in sample input (50 – 1,500 µL plasma extract), which is not likely to occur in ordinary experiments. Under typical experimental conditions with the same chromatographic system and equal input across all samples, the Workflow including Dual MSTUS normalization can be expected to improve insight generation with greater biological significance and statistical significance.

## Discussion

We present an IROA-based Dual MSTUS Workflow that is applicable across chromatographic systems, ion sources, and biological matrices. The Workflow, including suppression correction and Dual MSTUS normalization algorithms, facilitates accurate comparison of metabolomic profiling data across: i) unclean and clean ionization sources; ii) IC-, HILIC-, and RPLC-MS systems; and iii) plasma and urine matrices. Overall, the Workflow provides an almost universal antidote to the negative effects of ion suppression across all analytes in a non-targeted metabolite profiling study. By doing so, it can increase the sensitivity, accuracy, precision, and biological and statistical significance of such measurements.

The IROA Workflow has significant implications for the metabolomics field. Matrices like fine-needle aspirates and stool, which produce high, variable suppression can benefit most profoundly. Fine-needle aspirates (FNAs) are central to the diagnosis and management of breast cancer, and stool profiling is fundamental to microbiome metabolite profiling. Notably, uncleaned MS system components variably affect intra- and inter-instrument performance within and across laboratories, but the IROA Workflow produces equivalent data for equivalent samples processed across variable conditions. Since the very high attrition rate for clinical biomarker development is thought to be associated with hidden artifacts in the source data, inappropriate statistical methods, and unmanageable variation[29,30], the improved rigor and reproducibility provided by the IROA Workflow could decrease metabolomics-based biomarker failure.

The Workflow is highly effective but does have limitations. Chief among them is that the output includes only metabolites that are detected in both the $^{12}C$ and $^{13}C$ channels. One factor that can limit such detection is instrument sensitivity. Another factor is ion suppression. A third factor is low abundance in the IROA-IS. Ongoing advances in MS sensitivity and in the IROA-IS itself will translate into increased coverage of the metabolome and increased utility of the IROA TruQuant workflow.

By addressing multiple sources of error, the IROA Workflow effectively corrects for a majority of the intrinsic inaccuracies of MS analyses and could help to move the metabolomics field toward standardization of non-targeted analyses.

## LIST OF ABBREVIATIONS

IC: Ion Chromatography
IROA: Isotopic Ratio Outlier Analysis
IROA-IS: IROA-Internal Standard
IROA-LTRS: IROA-Long Term Reference Standard
MSTUS: Mass Spectrum Total Useful Signal
HILIC: Hydrophilic Interaction Liquid Chromatography
RPLC: Reverse Phase Liquid Chromatography
NF: Normalization Factor
%CV: Percent Coefficient of Variations
12C SC: 12C suppression corrected
AUC: Area under the curve

## Methods

### Sample preparation

For each chromatographic system tested, triplicate 225 µL aliquots of human plasma with K2-EDTA as the anticoagulant (BioIVT lot #BRH1225104) were added to separate 15 mL polypropylene centrifuge tubes and combined with 5.4 mL dry ice-cooled methanol, and centrifuged at 16,100 *g* at 4 °C for 10 minutes. The supernatant was transferred to a new tube from which aliquots of 1500, 1250, 1000, 750, 500, 250, 100, and 50 µL were prepared. The methanol extracts were then dried using a centrifugal vacuum concentrator and stored at -80°C. Immediately before analysis, samples were reconstituted in 40 µL of IROA-IS solution, prepared by dissolving the contents of an IROA-IS vial in 1.2 mL $H_2O$, vortexing, and briefly centrifuging. Following the IROA TruQuant protocol (**Fig. 2**), the entire contents of the tubes were then transferred to polypropylene autosampler vials. IROA-LTRS, an isotopically-labeled complex standard composed of extracts universally labeled at both 5% and 95% U-13C in a 1:1 ratio, was prepared by dissolving the contents of an IROA-LTRS vial in 40 µL $H_2O$. In addition to blank and process blank samples (deionized $H_2O$, before and after SOP), injections of the 350 µL aliquot plasma extract sample (without IROA-IS) and the IROA-IS solution alone served as controls. A 3 µL injection of IROA-LTRS and 5 µL injection of each sample were analyzed.

### Reverse Phase Liquid Chromatography (RPLC)

RPLC mobile phase A (MPA; weak) was water containing 10 mM ammonium acetate and 0.1% formic acid, and mobile phase B (MPB; strong) was methanol containing 10 mM ammonium acetate and 0.1% formic acid. The stationary phase was a Phenomenex Kinetex C18 column (2.6 µm particle size, 100 Å, 150 x 2.1 mm) with column compartment kept at 40°C. The autosampler tray was chilled to 4°C. The mobile phase flow rate was 200 µL/min, and the gradient elution program was: 0-5 min, 5% MPB; 5-30 min, 5-95% MPB; 30-40 min, 95% MPB; 40-45 min, 5% MPB. The total run time was 60 min.

### Hydrophilic Interaction Liquid Chromatography (HILIC)

HILIC mobile phase A (MPA) was 95/5 (v/v) water/acetonitrile containing 20 mM ammonium acetate and 20 mM ammonium hydroxide (pH~9), and mobile phase B (MPB) was acetonitrile. Thermo Vanquish LC system included a Xbridge BEH Amide column (3.5 µm particle size, 100 x 4.6 mm) with column compartment kept at 30°C. The autosampler tray was chilled to 4°C. The mobile phase flow rate was 300 µL/min, and the gradient elution program was: 0-3 min, 85% MPB; 3-10 min, 85-30% MPB; 10-20 min, 30-2% MPB; 20-25 min, 2% MPB; 26-30 min, 2-85% MPB. The total run time was 30 min.

### Ion Chromatography (IC)

IC mobile phase A (MPA; weak) was water, and mobile phase B (MPB; strong) was water containing 100 mM KOH. A Thermo Scientific Dionex ICS-6000+ system included a Thermo IonPac AS11 column (4 µm particle size, 250 x 2 mm) with column compartment kept at 35°C. The autosampler tray was chilled to 4°C. The mobile phase flow rate was 360 µL/min, and the gradient elution program was: 0-5 min, 1% MPB; 5-25 min, 1-35% MPB; 25-39 min, 35-99% MPB; 39-49 min, 99% MPB; 49-50, 99-1% MPB. The total run time was 50 min. To assist the desolvation for better sensitivity, methanol was delivered by an external pump and combined with the eluent via a low dead volume mixing tee.

### Ultra-high resolution mass spectrometry

For HILIC and RPLC chromatographic systems, a Thermo Scientific Orbitrap Fusion Lumos Tribrid mass spectrometer was operated in full scan mode using a scan range of 70-1,400 *m/z* and a resolution of 240,000. Heated electrospray positive ionization used a spray voltage of 3,500 (V) and vaporizer and capillary temperatures of 250 and 375°C, respectively. The sheath, auxiliary, and sweep gas pressures were 35, 10, and 0 (arbitrary units), respectively. Identical parameters were used for negative ionization with the exception of -2,800 (V) spray voltage. For IC-MS, data were acquired using a Thermo Orbitrap IQ-X Tribrid Mass Spectrometer under ESI negative ionization mode with settings otherwise as noted above.

### Data processing and calculations

Raw data files acquired by XCalibur software (Thermo Fisher) were converted to mzXML format using ProteoWizard's msConvert (version 3.0.193336, 64 bit) using only the "peak picking by vendor" mode (both $MS^1$ and $MS^2$ scans were converted). The mzXML files were analyzed using ClusterFinder (version 4.2.21, 64-bit, IROA Technologies). IROA-LTRS mzXML files were analyzed in non-targeted mode to determine the identity, RT, and $MS^1/MS^2$ characteristics of the IROA-LTRS peaks. Those data were then used to create runtime libraries (Dual MSTUS Metabolite Lists), which were used as the basis for annotation and quantitative analyses of experimental samples. The quantitative data included raw, suppression-corrected, and normalized data for each peak in the analytical samples. Annotation data included the name of the compound, the reporting standard achieved, identity algorithm used (all inferred by comparison to the IROA-LTRS), retention time (RT), and m/z for both 12C and 13C monoisotopic peaks.

Calculations were performed automatically within ClusterFinder, using the algorithms described in the following sections.

### Quality control and quality assurance

The following additional sample types were included in the analysis: a) a distilled water blank, b) a sample with an aliquot level of 350 µL extract that did not contain the Internal Standard mixture, and c) a sample that contained the Internal Standard mixture but none of the experimental extract. The TruQuant Protocol also uses IROA-LTRS as a Quality Assurance that instrument performance is within acceptable levels and to aid in peak annotation under current runtime conditions. The use of the LTRS and these additional sample types are further described in the Results and Discussion section and in Supplemental Materials.

### Suppression-correction calculation

As part of the experimental design ion suppression was intentionally induced by increasing sample concentration. The following algorithm was employed for the correction of ion suppression.

**Equation 1**: $[AUC\text{-}12C_{suppressed}] / [AUC\text{-}13C_{suppressed})$ = $[AUC\text{-}12C_{suppression\text{-}corrected}]/[AUC\text{-}13C_{suppression\text{-}corrected}]$. In **Equation 1**, the raw MS data provide values for $[AUC\text{-}12C_{suppressed}]$ and $[AUC\text{-}13C_{suppressed}]$. $[AUC\text{-}13C_{suppression\text{-}corrected}]$ can be approximated from the least suppressed value in the data set—the Internal Standard Only (IS-ONLY), internal standard sample. For optimal accuracy, it is critical that the AUCs include the sum of all of the isotopologs derived from the sample (12C end of the isotopolog ladder) and the internal standard (13C end of the isotopolog ladder). Whereas the sample isotopologs are generally limited to the 12C monoisotopic peak (M) and its M+1, the IS will include the 13C monoisotopic peak (U-13C) and all of its isotopologs (M-1, M-2, etc.). We can solve **Equation 1** for [AUC-$12C_{suppression\text{-}corrected}$].

It is important to note that other values can be used in place of the "least suppressed value" to estimate [AUC-$13C_{suppression\text{-}corrected}$]. For instance, if the concentration of the internal standard is held constant across experiments performed over a period of time, then the least-suppressed value could be a historically and more accurately determined value. The least-suppressed value could be either a quantitative molar quantity or a relative intensity (e.g., an AUC). The values determined from **Equation 1** can be used to calculate % suppression as (1-[AUC-$12C_{suppressed}$]/[AUC-$12C_{suppression\text{-}corrected}$]).

**Dual MSTUS algorithm**

We developed a modified version of the mass spectrometry total useful signal (MSTUS) algorithm as follows[28]. Because it uses two MSTUS values, i.e. that of the IROA-IS sample ([MSTUS-13C]) and the original natural abundance sample ([MSTUS-12C]), we refer to this as a "Dual MSTUS algorithm" (see Results and Discussion).

Step 1: Calculate [MSTUS-12C] and [MSTUS-13C].

The Dual MSTUS Metabolite List for each sample includes only those peaks that are found in both the sample ($^{12}C$) and IROA-IS ($^{13}C$).

[MSTUS-12C] = the sum of the [AUC-12C suppression-corrected] values for all 12C peaks that are paired with a 13C peak.

[MSTUS-13C] = the sum of the [AUC-13C suppression-corrected] values for all IS peaks that are paired with a 12C peak.

Step 2: Calculate the sample-specific Normalization Factor (NF).

**Equation 2:** NF = [MSTUS-12C] / [MSTUS-13C]

NF considers the total peak intensity in the sample relative to the total peak intensity in the IROA-IS to adjust the total content of the sample to the total content of the internal standard. Leveraging the statistical principal that variance decreases with increasing sample size, greater accuracy is achieved when a larger number of metabolites is used to establish the total content of each sample.

Step 3: Calculate normalized values for each metabolite in each sample.

Once NF has been determined for each sample, each [AUC-12C suppression-corrected] value (**Equation 1**) is normalized by dividing by NF as indicated in **Equation 3**. The calculated NF values were extremely close to the values expected from the known aliquot sizes (**Fig. 6**).

**Equation 3**: [AUC-12C normalized] = [AUC-12C suppression-corrected] / NF.

The process for Dual MSTUS normalization is straightforward. It involves two main steps. First, NF is computed for each sample (**Equation 2**) based on the Dual MSTUS Metabolite List, which includes peaks found in both the $^{12}C$ and $^{13}C$ channels of each sample. Second, the suppression-corrected $^{12}C$ values (**Equation 1**) of each metabolite in the Dual MSTUS Metabolite List are adjusted so that their sum is equal to the total [MSTUS-13C] value. That adjustment aligns with the original MSTUS rationale that normalizing over many metabolites will average out experimental variance, improving our ability to analyze and interpret the underlying biology accurately. By first suppression-correcting then applying the dual MSTUS NF, multiple sources of variability can be mitigated to provide greater rigor and reproducibility in non-targeted metabolomics. The total number of metabolites detected in each experimental condition determine the normalized linear dynamic range.

## Data availability

All data are available in the article, Supplementary Information, and source data. Source data are provided with this paper.

## AUTHOR INFORMATION

Corresponding Author

Philip L. Lorenzi, The University of Texas MD Anderson Cancer Center, 7435 Fannin Street, Room 2SCR3.3029, Unit 0950, Houston, TX 77054. Phone: 713-792-9999. Email: PLLorenzi@mdanderson.org.

Author Contributions

Experimental design was performed by IM, LV, CB, and PLL. Data acquisition was performed by LT, BW, SM, BT, and LV. Data analysis and interpretation were performed by IM, LV, AR, FDJ, and CB. Manuscript composition was done by IM, LV, CB, and PLL. Manuscript review and editing were done by IM, JNW, RA, FDJ, CB, and PLL.

**Notes**
**The authors IM, BW, LV, LT, SM, BT, RA, JNW, and PLL declare no competing financial interest. CB, FDJ, and AR are employed by IROA Technologies.**

## ACKNOWLEDGMENT

The Metabolomics Core Facility is supported by NIH grants S10OD012304-01 and P30CA016672.

## Main Figures 1-6

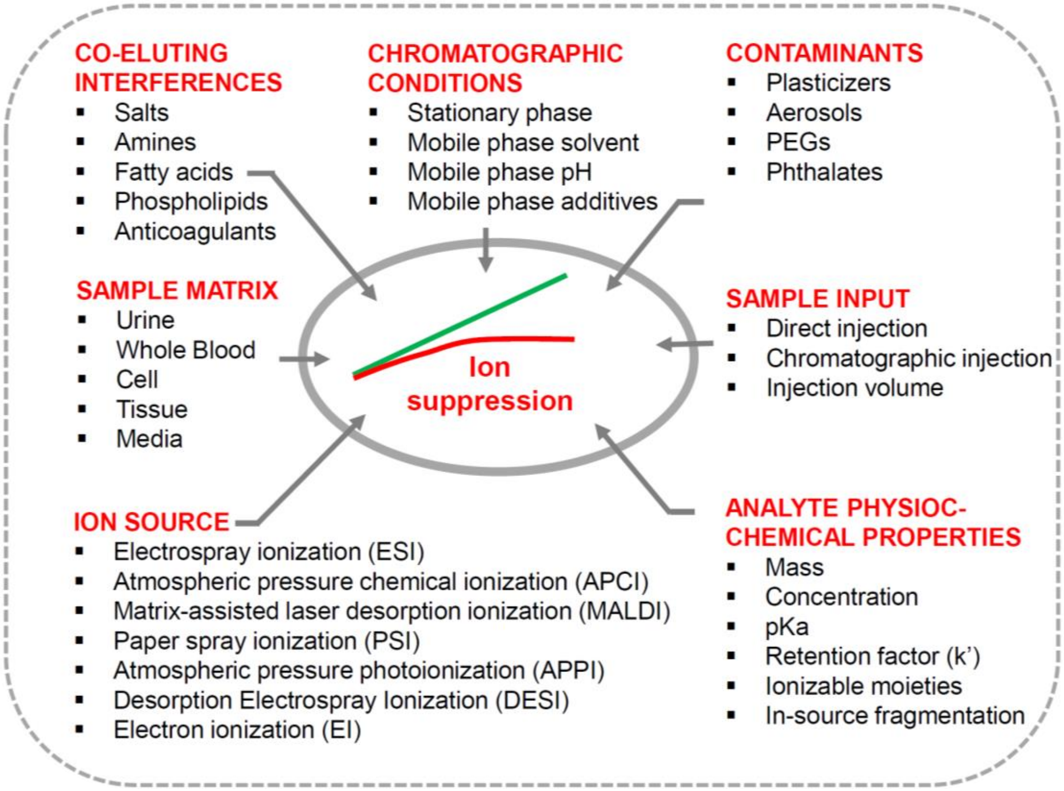

**Fig. 1 | Pre-analytical and analytical variables that affect quantitative rigor and reproducibility in MS-based metabolomics.** The red line represents observed, suppressed signal, which can be caused by the listed factors. The green line represents suppression-corrected signal, which can be realized by the techniques described in this paper. Representative examples of factors that affect the quantitative accuracy of MS-based measurements are listed and have been reviewed elsewhere[12,13].

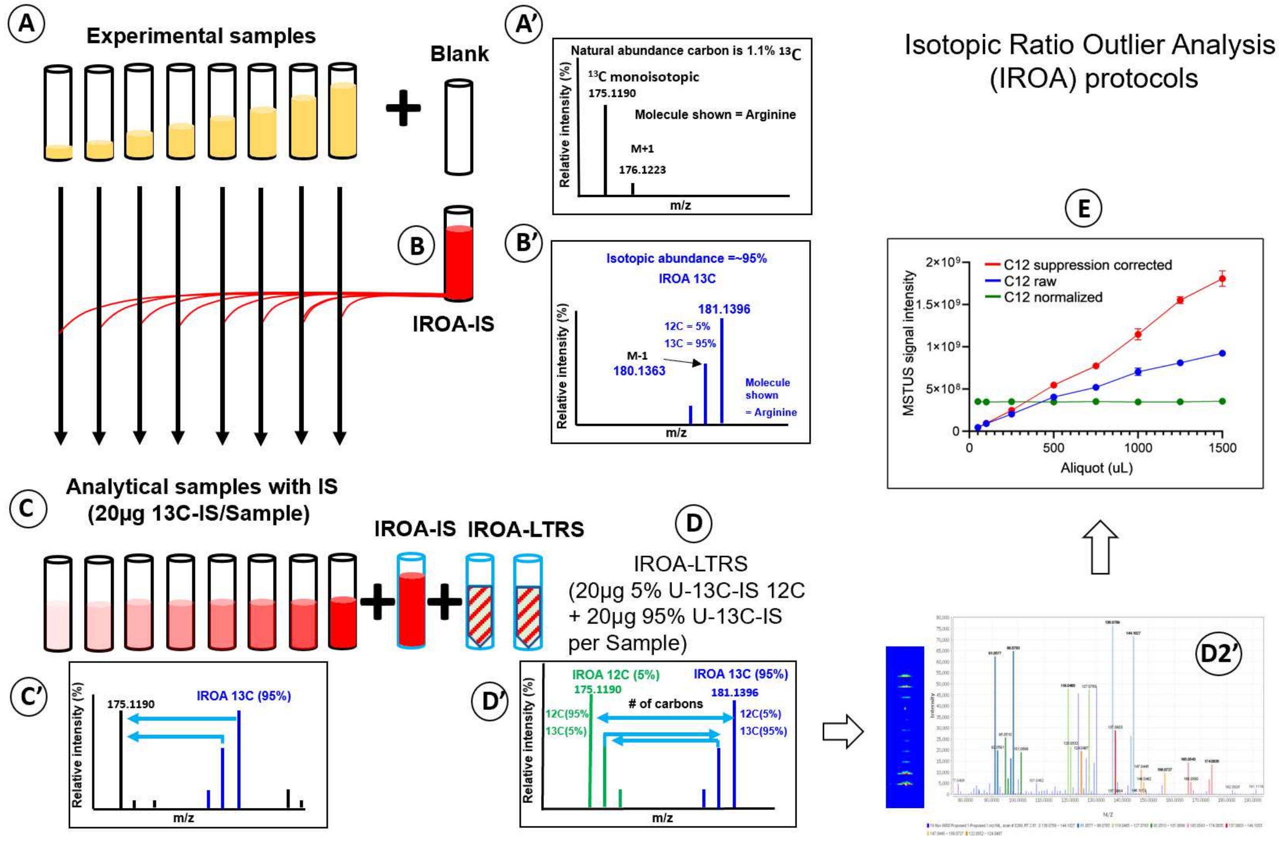

**Fig. 2 | IROA TruQuant Workflow.** In this protocol the experimental samples (**A**) are prepared and dried. They are then reconstituted with a solvent containing the IROA-IS (**B**) to yield the analytical samples (**C**). The analytical samples are randomized and injected within a sequence that starts and ends with injections of the IROA-LTRS (**D**), which is also injected approximately every 10 injections. Based on the presence of the IROA-IS, each sample can be suppression-corrected and normalized despite significant differences in sample input (original sample aliquot volume prior to dry down) (**E**).

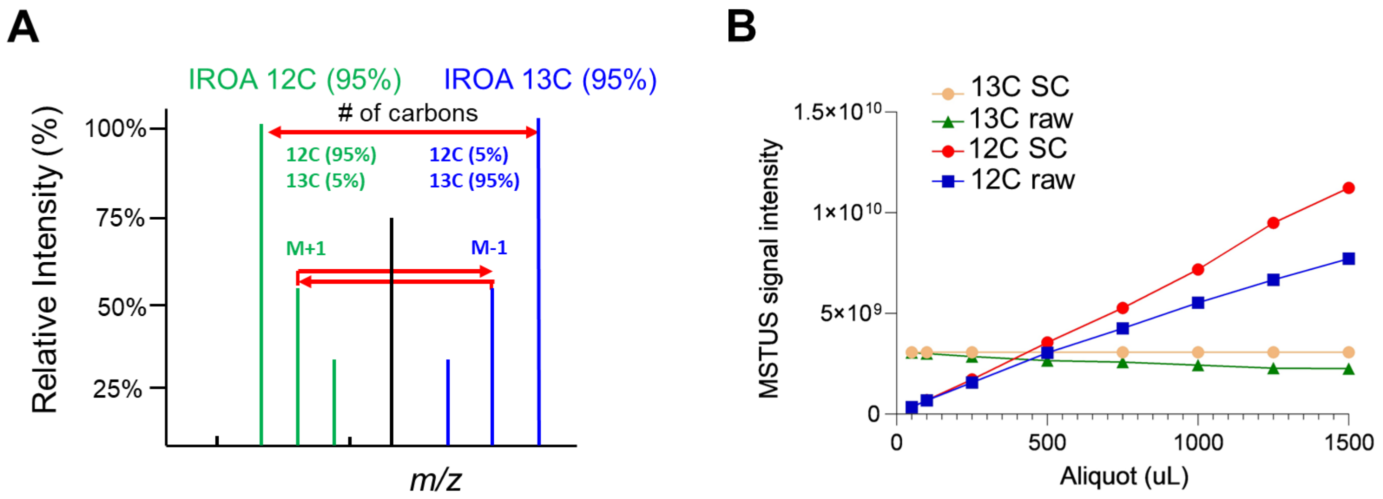

**Fig. 3 | Identification of IROA metabolite peaks.** (A) Detection and identification of metabolites by their isotopolog ladder patterns in IROA-LTRS. (B) Calculation of MSTUS values was performed as described in the Methods section. The illustrated data are from RPLC-MS with cleaned ionization source. 13C SC = 13C suppression corrected MSTUS; 13C raw = 13C raw MSTUS; 12C SC = 12C suppression corrected MSTUS; 12C raw = 12C raw MSTUS.

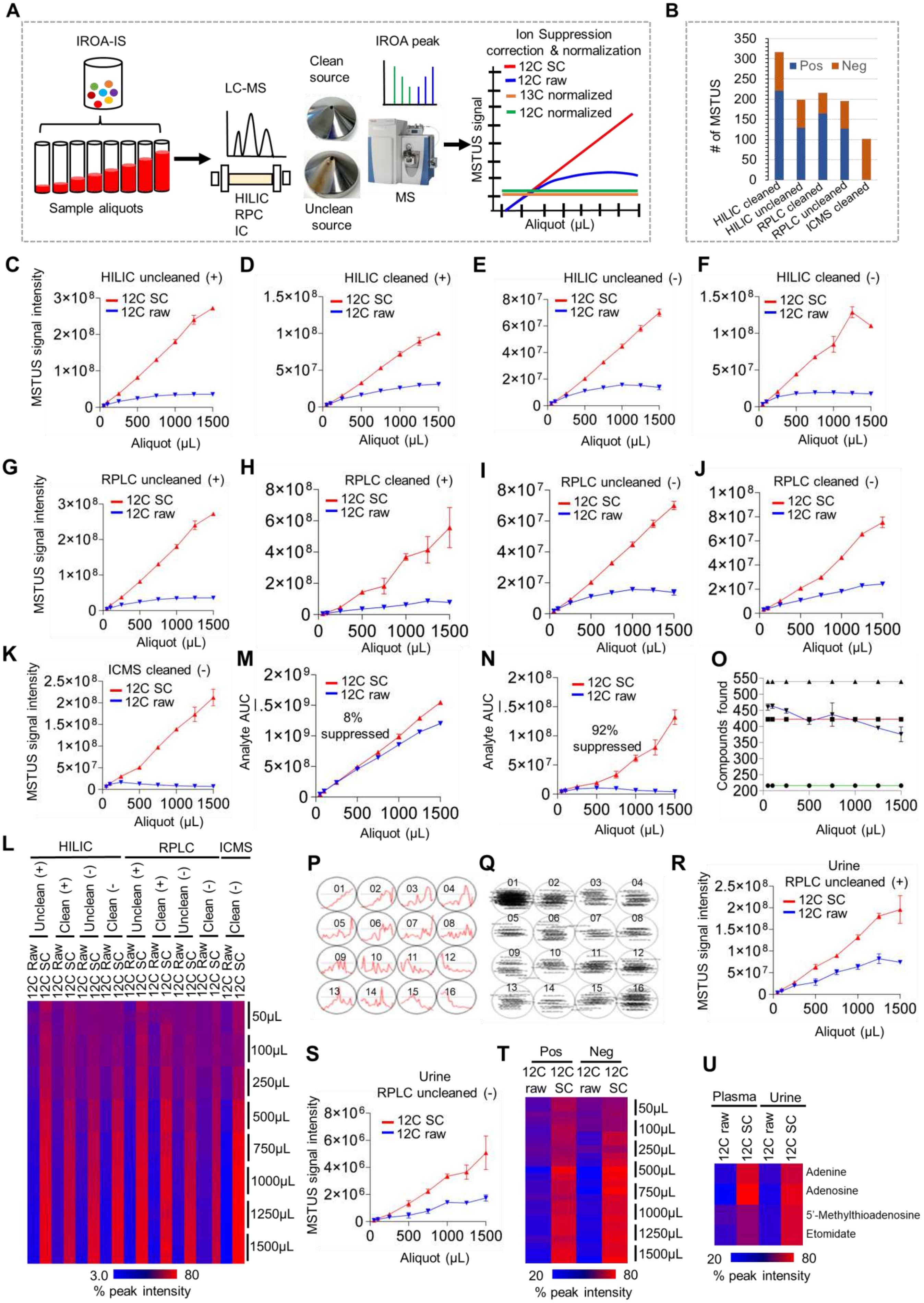

A
IROA-IS
Sample aliquots
LC-MS
HILIC
RPC
IC
Clean source
Unclean source
IROA peak
MS
Ion Suppression correction & normalization
12C SC
12C raw
13C normalized
12C normalized
MSTUS signal
Aliquot (μL)
B
# of MSTUS
Pos
Neg
HILIC cleaned
HILIC uncleaned
RPLC cleaned
RPLC uncleaned
ICMS cleaned
C
HILIC uncleaned (+)
D
HILIC cleaned (+)
E
HILIC uncleaned (-)
F
HILIC cleaned (-)
G
RPLC uncleaned (+)
H
RPLC cleaned (+)
I
RPLC uncleaned (-)
J
RPLC cleaned (-)
K
ICMS cleaned (-)
MSTUS signal intensity
M
8% suppressed
Analyte AUC
N
92% suppressed
O
Compounds found
L
HILIC
RPLC
ICMS
Unclean (+)
Clean (+)
Unclean (-)
Clean (-
Unclean (+)
Clean (+)
Unclean (-)
Clean (-)
Clean (-)
12C Raw
12C SC
50μL
100μL
250μL
500μL
750μL
1000μL
1250μL
1500μL
3.0
80
% peak intensity
P
Q
R
Urine RPLC uncleaned (+)
S
Urine RPLC uncleaned (-)
T
Pos
Neg
12C raw
12C SC
20
U
Plasma
Urine
Adenine
Adenosine
5'-Methylthioadenosine
Etomidate

**Fig. 4 | Ion suppression correction by IROA-IS across chromatographic systems and conditions.** (A) The IROA ion suppression correction workflow. (**B**) Number of MSTUS peaks detected across analytical conditions. (**C-K**) Raw MSTUS-12C (blue lines) and suppression-corrected MSTUS-12C (red lines) values are shown for: (**C**) HILIC positive mode, uncleaned source; (**D**) HILIC positive mode, clean source; (**E**) HILIC negative mode, uncleaned source; (**F**) HILIC negative mode, clean source; (**G**) RPLC positive mode, uncleaned source; (**H**) RPLC positive mode, clean source; (**I**) RPLC negative mode, uncleaned source; (**J**) RPLC negative mode, clean source; and (**K**) IC negative mode, cleaned source. (**L**) Ratio of raw MSTUS-12C to suppression-corrected MSTUS-12C peak intensity across chromatographic methods and experimental conditions. (**M**) Raw and suppression-corrected tryptophan values in RPLC positive ionization mode with cleaned source. (**N**) Raw and suppression-corrected pyroglutamic acid values in IC negative ionization mode. (**O**) Identified chemical composition in entire RPLC clean dataset, as an example. (**P**) Kohonen Self Organizing Maps (SOM) show suppression patterns in the RPLC Clean raw data set for all 539 compounds. (**Q**) Density map show compounds associated with each of the patterns discovered in Fig. O. (**R**) Raw MSTUS-12C (blue lines) and suppression-corrected MSTUS-12C (red lines) values are shown for RPLC positive mode and RPLC negative mode (**S**) for urine. (**T**) Ratio of raw MSTUS-12C to suppression-corrected 12C peak intensity for urine matrix in positive and negative ion modes. (**U**) Plasma and urine MSTUS-12C signals for 4 common metabolites before and after suppression correction. 12C SC =12C suppression corrected MSTUS; 13C raw = 13C raw MSTUS.

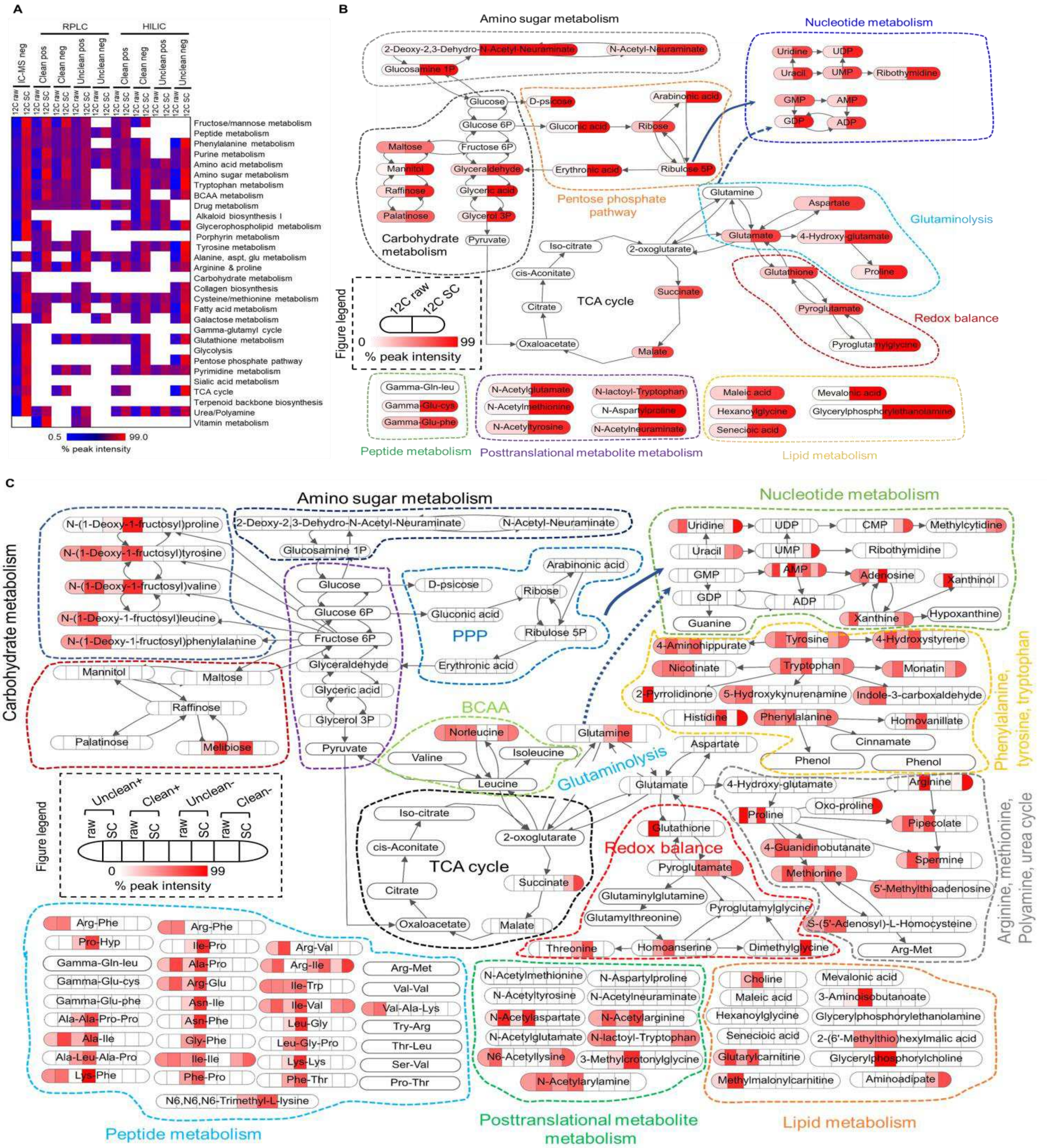

A
RPLC
HILIC
IC-MS neg
Clean pos
Clean neg
Unclean pos
Unclean neg
12C raw
12C SC
Fructose/mannose metabolism
Peptide metabolism
Phenylalanine metabolism
Purine metabolism
Amino acid metabolism
Amino sugar metabolism
Tryptophan metabolism
BCAA metabolism
Drug metabolism
Alkaloid biosynthesis I
Glycerophospholipid metabolism
Porphyrin metabolism
Tyrosine metabolism
Alanine, aspt, glu metabolism
Arginine & proline
Carbohydrate metabolism
Collagen biosynthesis
Cysteine/methionine metabolism
Fatty acid metabolism
Galactose metabolism
Gamma-glutamyl cycle
Glutathione metabolism
Glycolysis
Pentose phosphate pathway
Pyrimidine metabolism
Sialic acid metabolism
TCA cycle
Terpenoid backbone biosynthesis
Urea/Polyamine
Vitamin metabolism
0.5
99.0
% peak intensity
B
Amino sugar metabolism
Nucleotide metabolism
Pentose phosphate pathway
Carbohydrate metabolism
Glutaminolysis
TCA cycle
Redox balance
Figure legend
12C raw
12C SC
0
99
% peak intensity
Peptide metabolism
Posttranslational metabolite metabolism
Lipid metabolism
C
Amino sugar metabolism
Nucleotide metabolism
Carbohydrate metabolism
PPP
BCAA
Glutaminolysis
TCA cycle
Redox balance
Phenylalanine, tyrosine, tryptophan
Arginine, methionine, Polyamine, urea cycle
Figure legend
Unclean+
Clean+
Unclean-
Clean-
raw
SC
0
99
% peak intensity
Peptide metabolism
Posttranslational metabolite metabolism
Lipid metabolism

**Fig. 5 | Global metabolic pathway analysis illustrating the effects of IROA ion suppression correction.** (**A**) Raw and suppression-corrected (SC) peak intensities across chromatographic systems and uncleaned and cleaned ionization source conditions. (**B**) Metabolic pathways determined by IC-MS before and after ion suppression correction. (**C**) Metabolic pathways determined by RPLC-MS before and after ion suppression correction. Data were drawn in SBGN (system biology graphical notation), Process Description (PD) and Activity Flow (AF) languages or Simple Interaction Format (SIF). Metabolites are color-coded based on the percent peak intensity. Raw = 12C raw; SC= 12C suppression corrected; Uncleaned+ = Uncleaned positive ion mode; clean+ = clean positive ion mode; uncleaned- = uncleaned negative ion mode; cleaned- = cleaned negative ion mode.

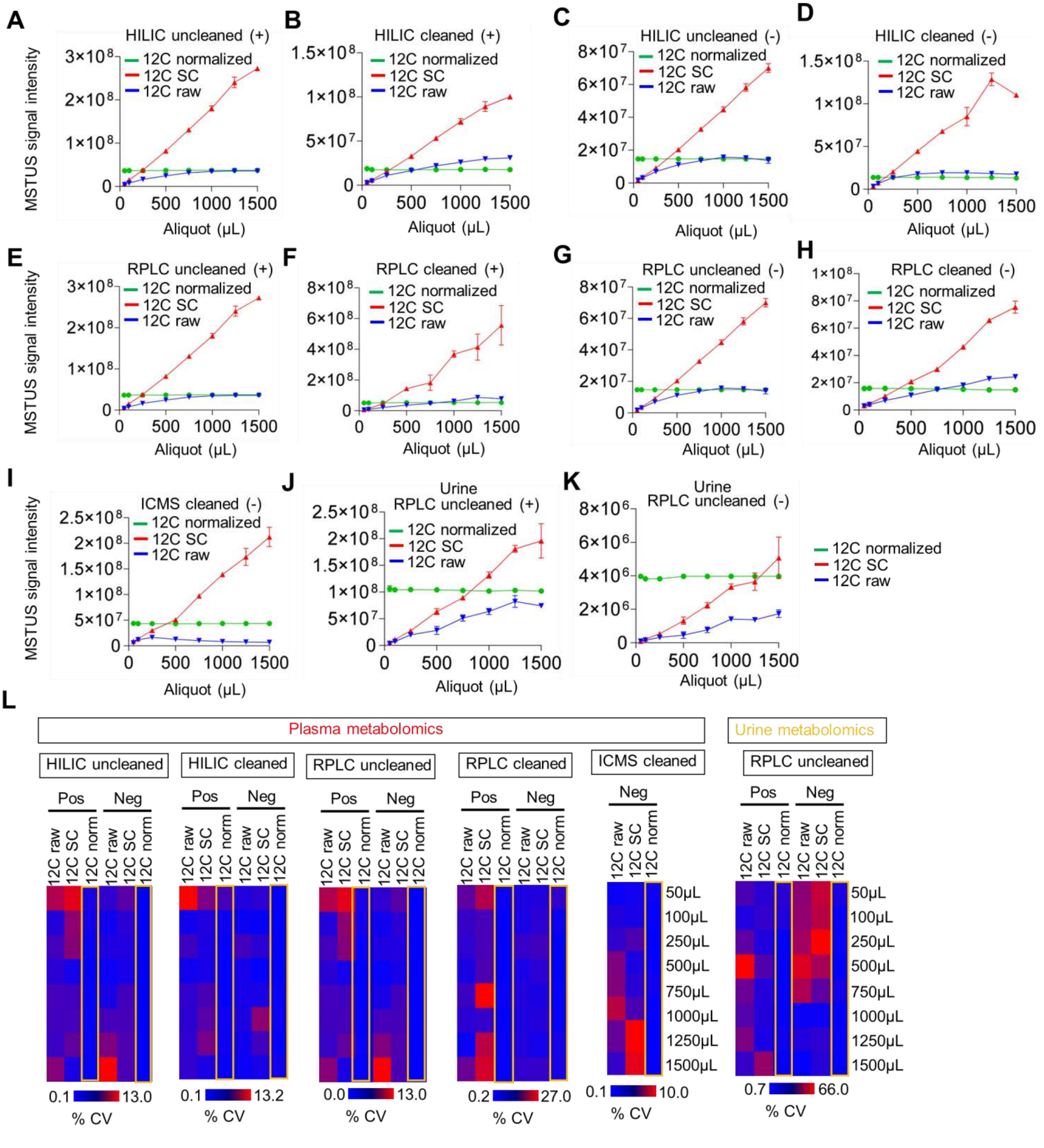

A
HILIC uncleaned (+)
B
HILIC cleaned (+)
C
HILIC uncleaned (-)
D
HILIC cleaned (-)
E
RPLC uncleaned (+)
F
RPLC cleaned (+)
G
RPLC uncleaned (-)
H
RPLC cleaned (-)
I
ICMS cleaned (-)
J
Urine
RPLC uncleaned (+)
K
Urine
RPLC uncleaned (-)
12C normalized
12C SC
12C raw
MSTUS signal intensity
Aliquot (μL)
L
Plasma metabolomics
Urine metabolomics
HILIC uncleaned
HILIC cleaned
RPLC uncleaned
RPLC cleaned
ICMS cleaned
RPLC uncleaned
Pos
Neg
12C raw
12C SC
12C norm
50μL
100μL
250μL
500μL
750μL
1000μL
1250μL
1500μL
0.1
13.0
0.1
13.2
0.0
13.0
0.2
27.0
0.1
10.0
0.7
66.0
% CV

**Fig. 6 | Dual MSTUS normalization standardizes metabolomic data across a broad range of analytical conditions.** Raw MSTUS-12C values (blue lines), normalized MSTUS-13C values (green lines), and suppression-corrected MSTUS-12C values (red lines) for indicated chromatographic systems and conditions in plasma. (**A** and **B**) HILIC positive mode, uncleaned and cleaned source; (**C** and **D**) HILIC negative mode, uncleaned and cleaned source; (**E** and **F**) RPLC (C18) positive mode, uncleaned and cleaned source; (**G** and **H**) RPLC (C18) negative mode, uncleaned and cleaned source; (**I**) IC-MS negative mode, clean source. Dual MSTUS normalization for urine by RPLC uncleaned positive mode (**J**) and negative mode (**K**). (**L**) Percent coefficient of variation (%CV) for raw, suppression-corrected, and normalized data from uncleaned and cleaned source conditions across different sample matrices and chromatographic systems including IC, HILIC, and RPLC.

## Supplementary Figures

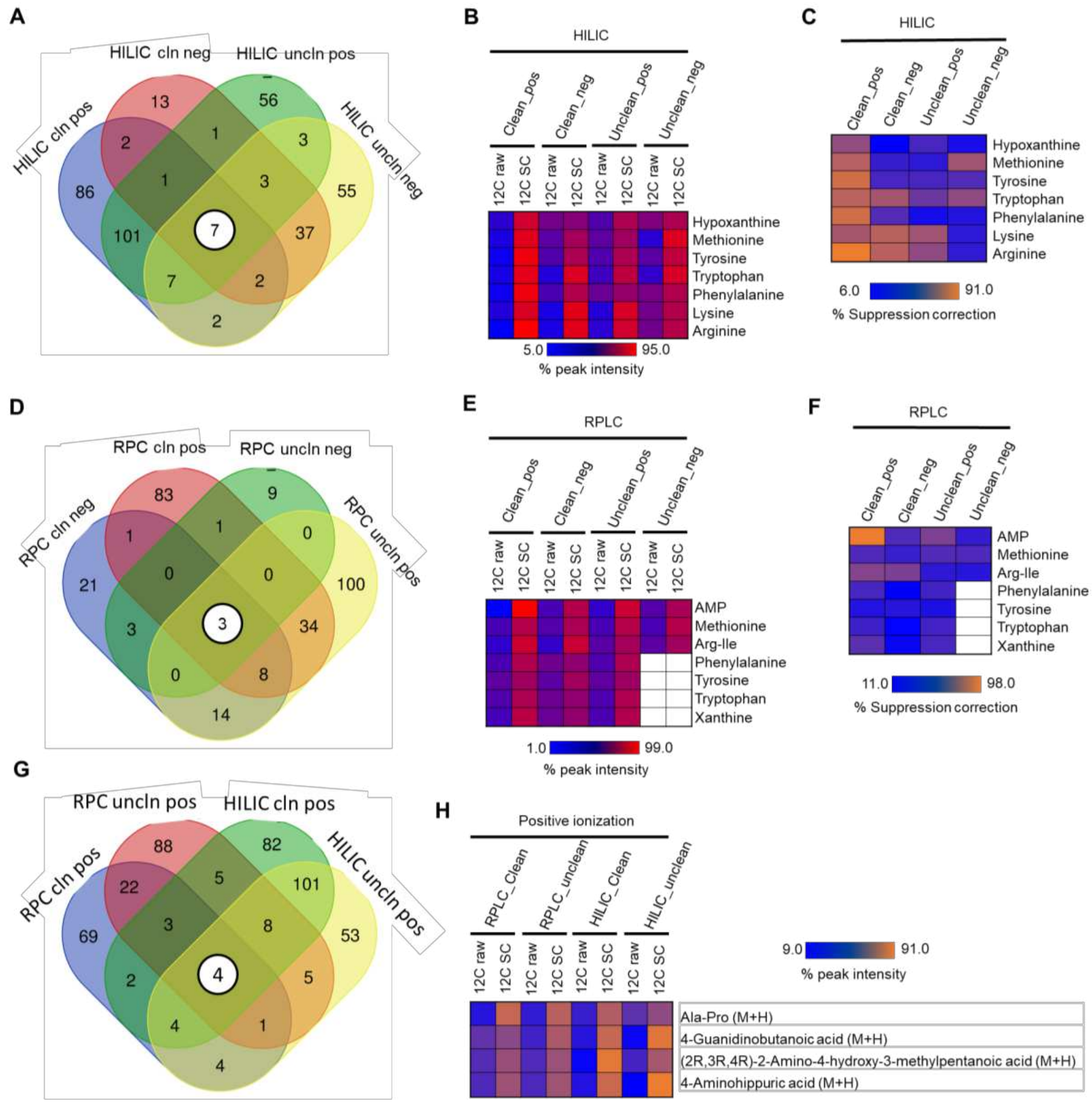

**Supplementary Fig. 1 | Assessment of IROA Workflow in ion suppression correction across chromatography.** (A) Commonly annotated 7 metabolites in different HILIC methods. (B) Raw MSTUS-12C and suppression-corrected 12C peak intensity for 7 metabolites that were commonly identified by HILIC. (C) Percent suppression correction among different HILIC methods by IROA Workflow. (D) Commonly annotated 3 metabolites in different RPLC methods. (E) Raw MSTUS-12C and suppression-corrected 12C peak intensity for 7 metabolites that were commonly identified by RPC. (F) Percent suppression correction among different RPC methods by IROA Workflow. (G) Commonly annotated 4 metabolites in RPLC and HILIC positive ion modes. (H) Raw MSTUS-12C and suppression-corrected 12C peak intensity for 4 metabolites that were commonly identified by both RPLC and HILIC chromatography in positive ion mode.

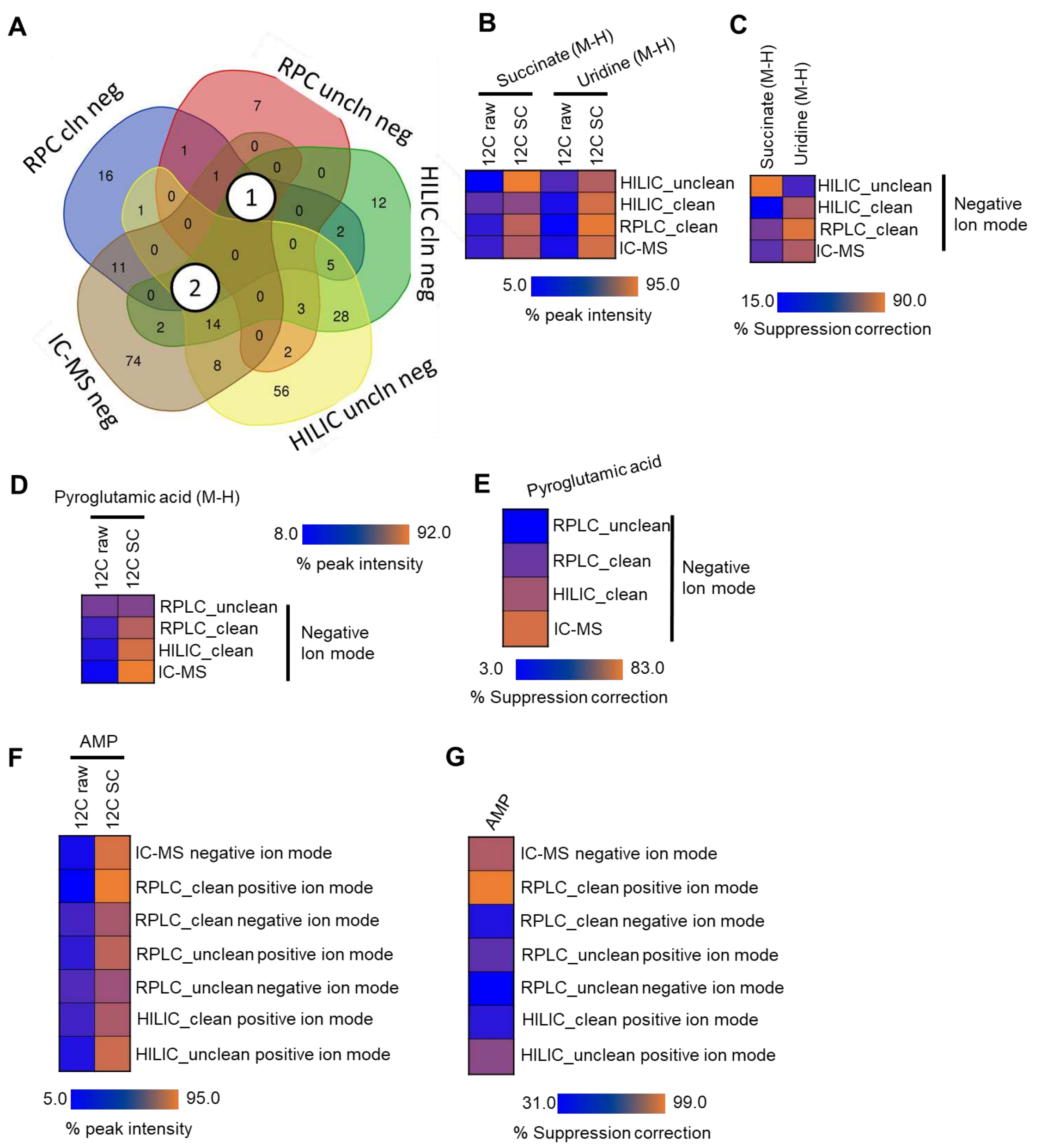

**Supplementary Fig. 2 | Assessment of IROA Workflow in ion suppression correction across ion modes of different chromatography.** (A) Commonly annotated metabolites in different ion mode of RPLC, HILIC, and ICMS. (B) Raw MSTUS-12C and suppression-corrected 12C peak intensity for succinate and uridine that were commonly identified by different chromatography. (C) Percent suppression correction among different chromatography modes by IROA Workflow. (D) Raw MSTUS-12C and suppression-corrected 12C peak intensity for pyroglutamic acid that was commonly identified by different chromatography. (E) Percent suppression correction of pyroglutamic acid among IC-MS, RPLC, and HILIC negative. (F) Raw MSTUS-12C and suppression-corrected 12C peak intensity for AMP that was commonly identified by different chromatography. (G) Percent suppression correction of AMP among IC-MS, RPLC, and HILIC methods.

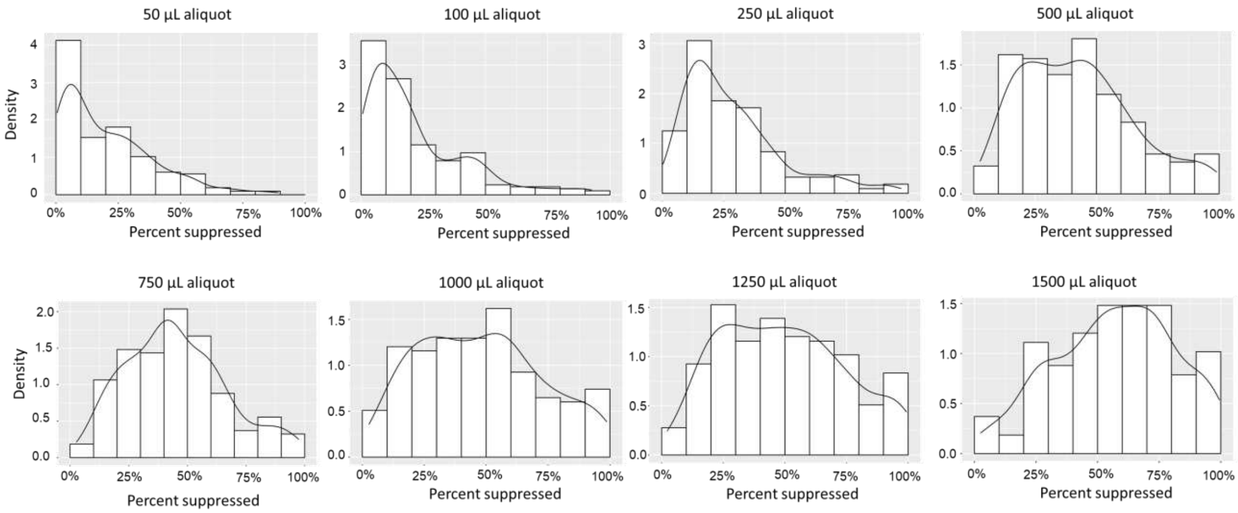

**Supplementary Fig. 3 | Distribution of suppression level seen in different aliquots.**

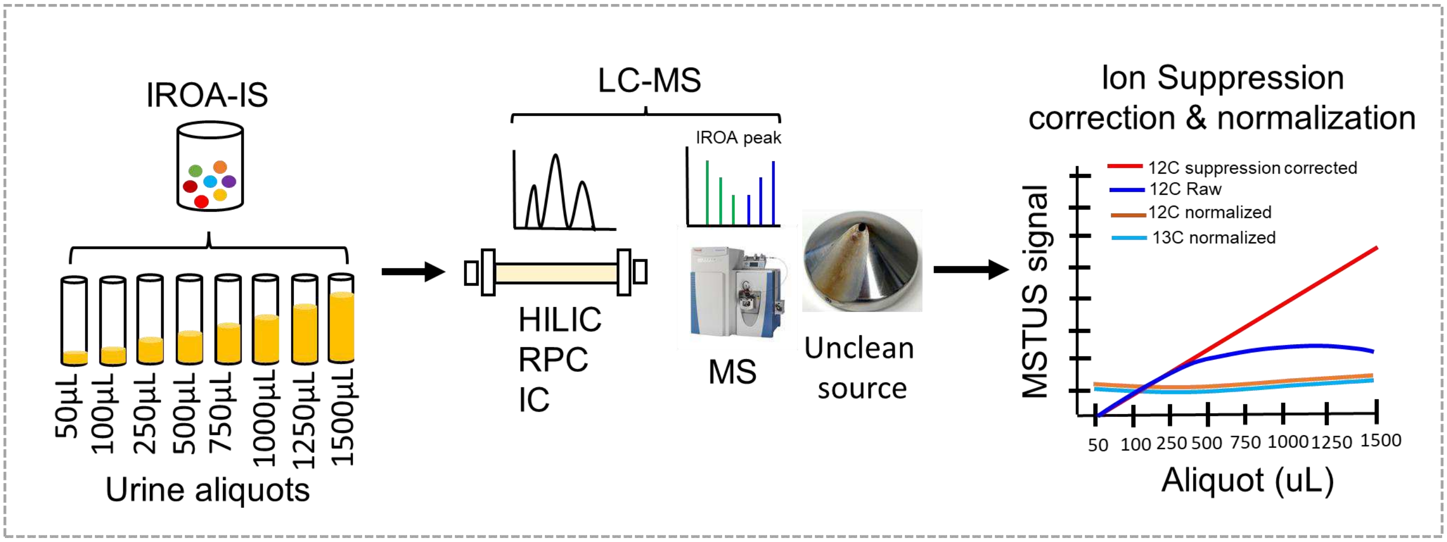

**Supplementary Fig. 4 | Ion suppression correction by IROA Workflow in urine metabolomics across RPLC uncleaned system with positive and negative ion modes.**

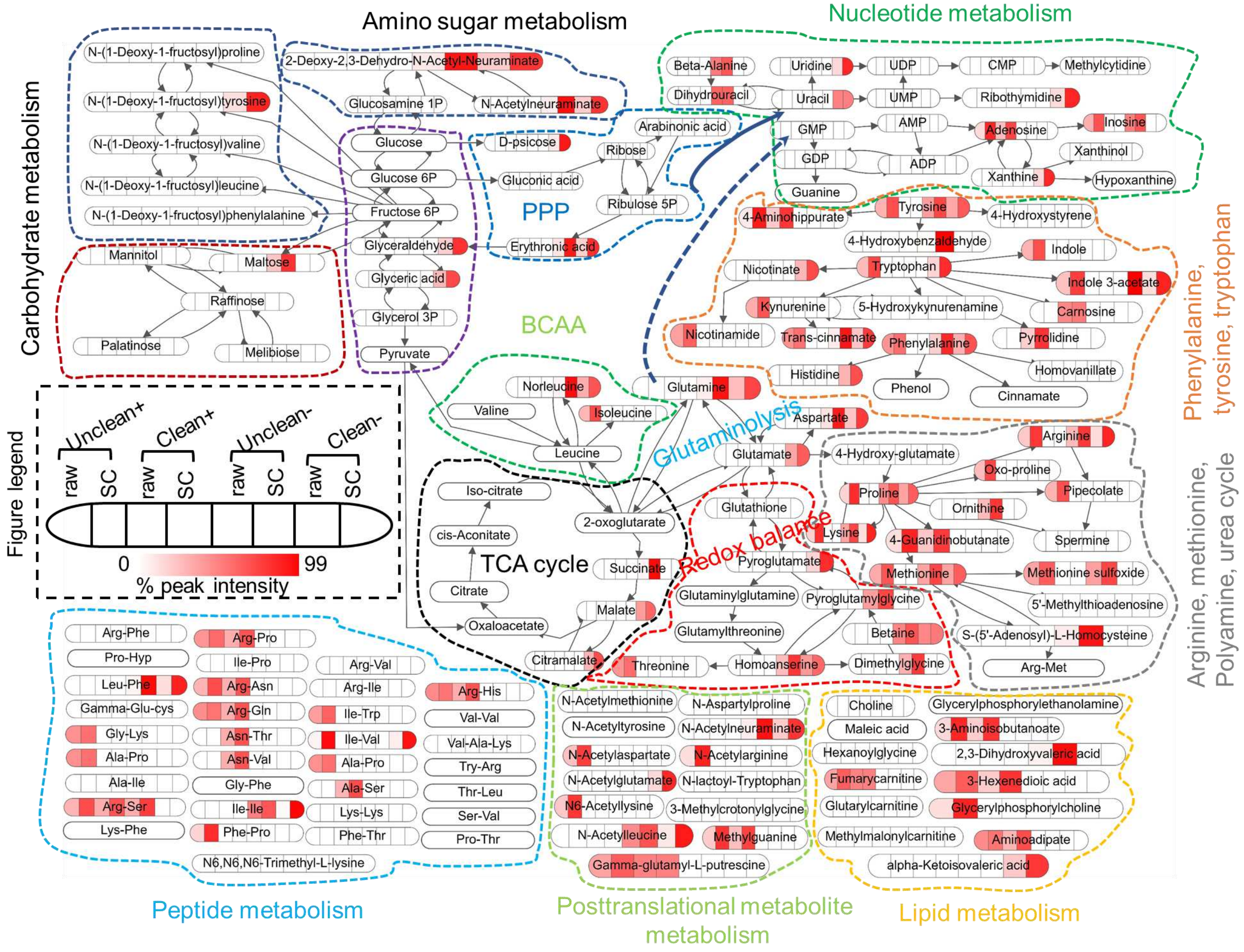

**Supplementary Fig. 5 | The IROA Workflow correct and recover peak intensities of global metabolic pathways analyzed by different HILIC chromatography.** Shown are metabolic pathways before (12C raw) and after ion suppression correction (12C suppression corrected) across different HILIC-based analytical methods and experimental conditions. Raw = 12C raw; SC= 12C suppression corrected; Uncleaned+ = Uncleaned positive ion mode; clean+ = clean positive ion mode; uncleaned- = uncleaned negative ion mode; cleaned- = cleaned negative ion mode. Data were drawn in SBGN (system biology graphical notation), Process Description (PD) and Activity Flow (AF) languages or Simple Interaction Format (SIF). Metabolites are color-coded based on the percent peak intensity.

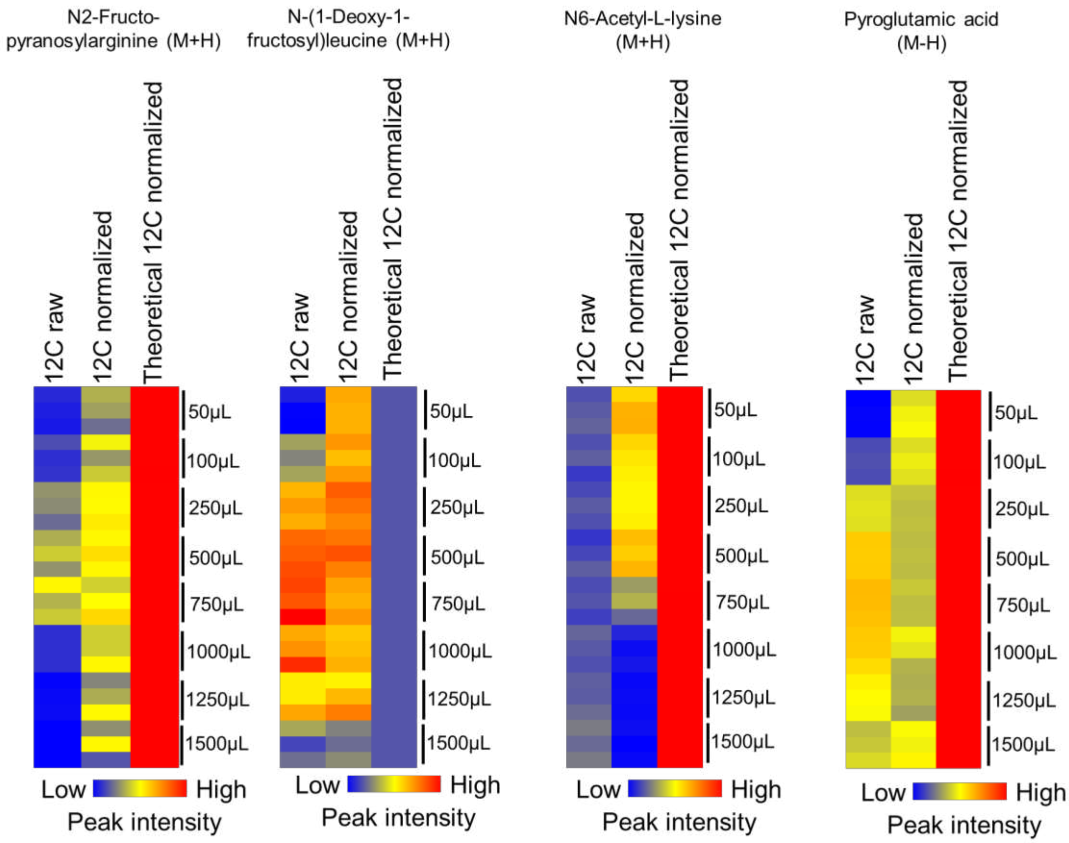

**Supplementary Fig. 6 | Normalization Factor (NF**) benefits metabolites that are not present in the IROA-IS. The application of the Normalization Factor (NF) to compounds that are not suppression corrected will normalize them but cannot correct for the ion suppression they have suffered. The normalized value is closer to the expected normalized value in all the analytes shown. Example analytes are shown from HILIC clean positive and negative mode data.
